# Common *FTO* rs9939609 variant and risk of type 2 diabetes in Palestine

**DOI:** 10.1186/s12881-018-0668-8

**Published:** 2018-08-31

**Authors:** Anas Sabarneh, Suheir Ereqat, Stéphane Cauchi, Omar AbuShamma, Mohammad Abdelhafez, Murad Ibrahim, Abdelmajeed Nasereddin

**Affiliations:** 1 Biochemistry and Molecular Biology Department, Faculty of Medicine, Al-Quds University, Abu Dis-East Jerusalem, Palestine; 2 CNRS, UMR8204, Lille, France; 3 INSERM, U1019, Lille, France; 4 Université de Lille, Lille, France; 5 Institut Pasteur de Lille, Centre d’Infection et d’Immunité de Lille, Lille, France; 6 Microbiology and immunology Department-Faculty of Medicine, Al-Quds University-Palestine, Abu Dis-East Jerusalem, Palestine; 7 Al-Quds Nutrition and Health Research Institute – Faculty of Medicine, Al-Quds University-Palestine, Abu Dis-Jerusalem, Palestine

**Keywords:** *FTO*, rs9939609 variant, T2DM, BMI, Palestine

## Abstract

**Background:**

Genetic and environmental factors play a crucial role in the development of type 2 diabetes mellitus (T2DM) and obesity. This study aimed to investigate the association of the fat-mass and obesity-associated gene (*FTO*) rs9939609 variant with T2DM and body mass index (BMI) among Palestinian population.

**Methods:**

A total of 399 subjects were recruited, of whom 281 were type 2 diabetic patients and 118 normoglycemic subjects. All of them were unrelated, aged > 40 years and recruited within the period 2016–2017. The A allele of FTO rs9939609 was identified by PCR–RFLP.

**Results:**

Significant association of the minor allele A of *FTO* rs9939609 and T2DM risk was observed with an allelic odd ratio of 1.92 (95% CI [1.09–3.29], *p* = 0.02) adjusted for age and gender, this association partly attenuated when adjusted for BMI with OR of 1.84, (95%CI [1.04–3.05], *p* = 0.03). Stratified data by glycemic status across *FTO* genotypes showed that A allele was marginally associated with increased BMI among diabetic group (*p* = 0.057) but not in control group (*p* = 0.7). Moreover, no significant association was observed between *FTO* genotypes and covariates of age, gender, T2DM complications or any tested metabolic trait in both diabetic and nondiabetic individuals (*p* > 0.05).

**Conclusions:**

The variant rs9939609 of the FTO gene was associated with T2DM in Palestine. This is the first study conducted on this gene in the Palestinian population and provides valuable information for comparison with other ethnic groups. Further analysis with larger sample size is required to elucidate the role of this variant on the predisposition to increased BMI in Palestinians.

## Background

Type 2 diabetes mellitus (T2DM) is the most common type of diabetes as it accounts for more than 90% of all diabetes cases worldwide (World Health Organization) [1]. Polymorphisms within the fat-mass and obesity-associated gene (*FTO*) are of particular interest as they have known effect on obesity which is a major risk factor for T2DM. A genome-wide association study (GWAS) conducted in 2007, confirmed that rs9939609 variant located within the first intron of the *FTO* gene predisposes European populations to diabetes through an effect on body mass index (BMI) [2, 3], while other reports from South Asian population showed that *FTO* gene variants increase the risk of type 2 diabetes independent of BMI [4]. Since then, several studies represent various ethnic populations, confirmed strong associations of the *FTO* rs9939609 with obesity [5, 6]. This association was not replicated in the Chinese Han population and African Americans [7, 8]. It is well-established that dyslipidemia is a risk factor for cardiovascular diseases (CVD) in diabetic patients. However, a study conducted by Doney et al. [9] demonstrated that A allele rs9939609 in the *FTO* gene increases the risk of myocardial infarction in patients with T2DM independent of BMI, glycated hemoglobin, mean arterial pressure and dyslipidemia. Moreover, a significant association of *FTO* variant was found in Indian patients with T2DM without dyslipidemia [10].

A sex-specific effect of *FTO* variants on susceptibility to obesity have been shown, a study -in 2016- indicated that the effect of *FTO* variants on T2DM susceptibility in Japanese men but not women is mediated through *FTO* effect on BMI [11]. In 2018, a case control study conducted on obese Iranian women showed that several *FTO* variants including rs9939609 were associated with T2DM and obesity as well [12]. A recent spatial and meta-analysis suggested a region-related associations between *FTO* rs9939609 and T2DM [13]. Thus, the reported results were not consistent in different ethnic population.

In Palestine, the prevalence of DM (for adults aged > 25 years) was 15.3% in 2010, but estimates have placed to be as high as 20.8% by 2020 [14, 15]. Diabetes and its complications are estimated to account for approximately 5.7% of all deaths in Palestine [16]. The prevalence of overweight and obesity is rapidly escalating in the youth and adults, probably due changes in lifestyle, further enhancing the risk of diabetes. In 2016, a cross sectional study among the students at An-Najah National University in Nablus district (North Palestine) showed that the prevalence of overweight and obesity was 26.2%, with significant increase in males (36.4%) compared to females (19.1%) [17]. Genetic association studies of T2DM among Palestinians are scarce. Two studies conducted by Ereqat and colleagues in 2009 [18, 19] investigated the genetic association of Pro12Ala Polymorphism of the PPAR-Gamma 2 gene and rs7903146 variant in the transcription factor 7 like 2 gene (*TCF7L2*) with T2DM. However, no studies have been conducted to determine the genetic association of *FTO* variants with T2DM and/ or obesity. Therefore, our study aimed to examine the association between the *FTO* rs9939609 SNP with the risk of T2DM and its-related phenotypes in Palestinian population.

## Methods

### Study population

A total of 399 unrelated individuals were recruited from different cities in Palestine. Two-hundred eighty one cases, aged > 40 years, were diagnosed by T2DM according to WHO criteria based on fasting plasma glucose 126 mg/dl and/or currently being treated with medication for diabetes. All participants were recruited within the period of 2016–2017 in collaboration with UNRWA clinics (Hebron and Ramallah, Palestine). The anthropometric measurements were collected from their medical records that included age, sex, family history, drug history, medical history and other related information.

Fasting blood was collected for biochemical tests and DNA studies. All the cases with probable diagnosis of type 1 diabetes were excluded. The control group (*n* = 118), was selected from individuals who came to the same clinic for health check-up with no past medical history for T2DM and no family history of diabetes in first-degree relations. Age at examination was > 40 years.

### Biochemical testing, DNA extraction and genotyping

Five milliliters of blood were obtained after overnight fast, collected in EDTA tubes, centrifuged at room temperature. Plasma glucose, cholesterol, HDL cholesterol, and triglyceride were determined by enzymatic methods as described by manufacturer’s instructions (Human, Wiesbaden, Germany). Genomic DNA was extracted from whole blood (300 μl) using genomic DNA purification kit QIAamp according to the manufacturer instructions (Qiagen, Hilden, Germany). DNA samples were stored at 4 °C for further analyses. Genotyping of the *FTO* rs9939609 SNP was done by PCR-based restriction fragment length polymorphism (RFLP) analysis as previously described [20] with the following modifications. The PCR reactions were carried out using 20 ng of purified genomic DNA samples, with 0.4 μM of the forward and reverse primers using PCR-Ready Supreme mix (Syntezza Bioscience, Jerusalem) in a final volume of 25 μl. The genotypes patterns were determined by 2% agarose gel electrophoresis (Agarose; Sigma-Aldrich, Munich, Germany) stained with Ethiduim bromide. A 5% masked, random sample of cases and controls were re-amplified and sent for sequencing to confirm the genotyping method.

### Statistical analysis

The genotype frequencies were tested for Hardy–Weinberg equilibrium using a chi-square test through the website http://www.oege.org/software/hwe-mr-calc.html. All statistical analysis was performed using SPSS v23.0 (SPSS, Chicago, IL). Pearson’s Chi-square test was used to compare allelic and genotypic frequencies between the diabetic and nondiabetic groups. ANOVA was used to assess the association between *FTO* genotypes and continuous variables. Logistic regression by R statistics (V 3.4.4) software was used to measure odd ratio (OR) for T2DM adjusted for age, gender and BMI.

## Results

### Biochemical characteristics of the study participants

The biochemical and anthropometric results of the 281 T2DM patients and 118 nondiabetic subjects are shown in Table 1. As expected, significant differences in biochemical parameters was observed between the two groups (*p* < 0.05). However, the mean total cholesterol was not significant between diabetic and non diabetic groups (*p* = 0.25). Among T2DM group, 64.4% (*n* = 181) were obese (BMI > 30 kg/m2), 30.2% (*n* = 85) were overweight (25–29.9 kg/m2) and 5.3% (*n* = 15) were nonobese (BMI < 30 kg/m2). Of them, 76.6% were treated with oral hypoglycemic agent, 25.3% received a combination of insulin and oral hypoglycemic agents and 2.1% were treated with insulin. Of these patients, 14.6, 12.1, 7.8 and 6% had cardio vascular disease (CVD), nephropathy, diabetic foot and retinopathy, respectively. Noteworthy, 75% of the cases had T2DM first-degree relatives. Among the control group, 44.1% (*n* = 52) were obese, 22.9% (*n* = 27) were overweight and 33.1% (*n* = 39) were not obese.

**Table 1:** Clinical and biochemical characteristics of case and control groups

	cases (*n*=281)	controls (*n*=118)	
Parameters			*P-value*
M:F ratio	94:187	48:70	
Age at diagnosis (years)	50.39+11.04	NA	
Age at sampling (years)	58.15±12.09	49.62±8.71	0.0001*
BMI	32.9±6.6	29.2±6.2	<0.0001*
SBP (mmHg)	136.21±17.42	122.33±11.44	0.0001*
DBP (mmHg)	80.21±10.83	76.72±9.37	0.0024*
FBS (mg/dl)	163.31±58.72	87.14±7.82	0.0001*
TC (mg/dl)	187.91±47.53	182.15±40.95	0.251
Treatment (OHA,C,I)(n)	204,71,6	NA	

Values are expressed as means ±SD, *P<0.05 is considered to be significant

*NA* Not applicable. Treatment at time of recruitment *OHA* oral hypoglycemic agent, *C* combination of insulin and hypoglycemic agent, *I* insulin

### Analysis of *FTO* variant

**Fig. 1:**
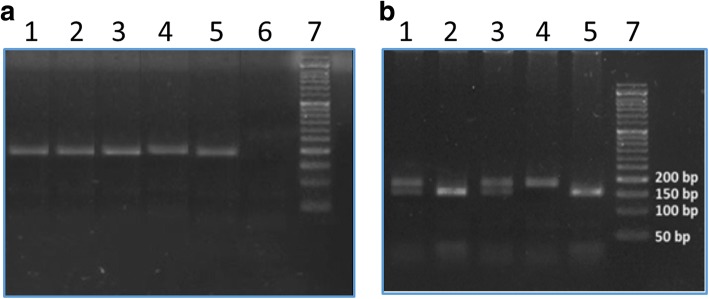
Agarose gel electrophoresis (2%) of *FTO* gene variant (**a**) PCR products showing 187 bp amplicon (Lanes 1–5), lane 6: Negative control, lane 7: 50 bp ladder. (**b**) Digested PCR product representing different genotypes: Lanes 1, 3: AT genotype; Lanes 2, 5: AA genotype; Lane 4: TT genotype; Lane 7: 50 bp ladder

*FTO* genotyping (rs9939609) was performed by PCR followed by RFLP. The presence of product was verified on a 2% agarose gel stained with ethidium bromide, a band of 187 bp was observed as shown in Fig. 1a. The PCR product was digested by *ScaI* restriction enzyme and visualized by 2% agarose gel. A band of 187 bp was observed for the TT genotype, two bands of 154, 33 bp was observed for the AA genotype while three bands of 187, 154, 33 bp were observed for the heterozygous genotype AT as shown in Fig. 1b.

### Association of *FTO* variant and T2DM

The genotype and allele frequency of *FTO* gene polymorphism (rs9939609) a;mong the two groups were analyzed and compared as shown in Table 2. Our results revealed that carriers of AA genotype was significantly higher in T2DM subjects compared to non diabetic individuals (36%) vs (16%))(*p* = 0.003). The genotyping distribution was in line of Hardy Weinberg equilibrium in all cases and controls (*p*= > 0.05). Logistic regression analysis was performed for AT and AA genotypes with TT as a reference genotype. We found that the AT genotype conferred 2.1 times higher risk for T2DM compared to TT genotypes unadjusted *p* < 0.0001 (Table 3). As our controls were younger than diabetic cases, logistic regression model adjusted for age and gender was used, and showed that allelic odd ratio was 1.92 (95% CI [1.09–3.29], *p* = 0.02). This association remained significant even after adjusting for age, gender and BMI (OR 1.84, 95%CI (1.04–3.05)). The highest risk was observed among AA carriers compared to those with TT genotypes (OR 4.03, 95% CI (2.01–8.06) *p* < 0.0001) as shown in Table 3.

**Table 2:** Allelic and genotypic frequency of *FTO* variant (rs9939609) among T2DM cases and controls

Genotype	cases(n)	Controls (n)	All Subjects (n)
TT	51	45	96
AT	129	54	183
AA	101	19	120
A allele (%)	58.8	38.9	53

**Table 3:** Association of *FTO* variant (rs9939609) with T2DM

Genotype	OR( 95% CI )	*P*-value	OR(95% CI)	*P-value
AA vs TT	4.69 (2.49–8.83)	<0.0001	4.03 (2.01–8.06)	<0.0001*
AT vs TT	2.11 (1.26-3.52)	<0.0001	1.84 (1.04-3.05)	0.034*
AA vs				
(AT+TT)	2.78 (1.72-4.49)	<0.0001	2.71 (1.5-4.9)	0.001*

*P values were from logistic regression models adjusted for age, gender and BMI, *P*<0.05 was considered significant

### Association of *FTO* variant with BMI

The entire data including all the study subjects (*n* = 399) was stratified based on *FTO* genotypes, a significant association was found between *FTO* genotypes and mean BMI, the AA genotypes had the highest BMI (33.29 ± 7.2), unadjusted *p* = 0.03. Because of potential confounding between T2DM and increased BMI as proxy measure of obesity, the data was stratified by glycemic status across *FTO* genotypes. Among diabetic group, a trend of increasing mean BMI was observed among the three genotypes: AA carriers had the highest BMI (34.11 ± 7.1) compared to AT (32.32 ± 6.1) and TT carriers (31.86 ± 6.5) but it was not significant (*p* = 0.057). However, this increasing trend was not found among the control group (*p* = 0.7) as shown in Table 4. Furthermore, no association was found between the *FTO* genotype and gender, age, plasma total cholesterol, as well as systolic and diastolic blood pressure among the two groups. Among diabetic group, no association was found between the *FTO* genotype and cardio vascular disease or diabetes complications (*p* > 0.05).

**Table 4:** Mean trait values stratified by glycemic status across *FTO* genotypes

	T2DM (*n*=281)				Control (*n*=118)			
	AA	AT	TT	*P-value	AA	AT	TT	*P-value
Number	101	129	51		19	54	45	
BMI (kg/m2)	34.11±7.1	32.32±6.1	31.86±6.5	**0.057**	28.89±6.96	29.77±6.1	28.75±5.8	0.688
SBP (mmHg)	133.94±16.9	138.26±16.4	135.47±20.4	0.166	119.3±16.3	124.2±10.7	121.4±9.5	0.222
DBP (mmHg)	78.68±11.9	80.94±10.1	81.35±10.5	0.206	72.8±9.2	76.7±10.2	78.5±8.1	0.087
FBS (mg/dl)	159.11±54.3	163.97±58.9	169.94±65.6	0.553	85.9±6.9	87.7±7.4	86.9±8.8	0.693
TC (mg/dl)	189.16±49.3	188.57±49.2	183.71±39.8	0.783	168.1±40.1	188.5±48.8	180.4±28.2	0.165

*P<0.05 was considered significant, obtained by ANOVA. Values are expressed as means ±SD. Bold number showed marginally significant association between A allele and increased BMI among diabetic group

## Discussion

To our knowledge, this study is the first to investigate the association of the *FTO* variant rs9939609 with type 2 diabetes and BMI in Palestine. The significance of common variants in the *FTO* gene for susceptibility to adiposity have been highlighted by large-scale studies among Europeans while conflicting results were reported in Asian populations [21, 22]. Our study showed a significant association of *FTO* variant rs9939609 with T2DM after adjustment by age and gender with an allelic odds ratio of 1.92 (95% CI [1.09–3.29], *p* = 0.02). Moreover, we noted that the association of *FTO* variant rs9939609 with T2DM was partially attenuated by adjusting for BMI with odd ratio of 1.84, 95%CI (1.04–3.05) *p* = 0.03, suggesting that the *FTO* -T2DM association was not completely mediated through *FTO* variant effect on BMI. Similar results were found in Indian, American and Chinese populations [4, 10, 23]. Vasan and colleagues [24] provided evidence that *FTO*-T2DM risk -among Asian Indians- was attenuated but not fully abolished when adjusting to BMI. In contrast, a recent study conducted in Kuwaiti population did not observe an association between the *FTO* rs9939609 with T2DM risk [25]. Two studies in North Indians and Asian Indians demonstrated a strong association of *FTO* rs9939609 with T2DM independent of BMI [4, 26]. However, contradictory results for association of *FTO* variants with T2DM have been reported in different ethnic groups of India [27]. Furthermore, a meta-analysis study conducted in South Asia showed that BMI and central obesity can partly account for the association of A allele of the *FTO* gene and diabetes, whereas this association was much reduced when adjusted for BMI in Europeans indicating ethnic-specific associations [28]. On the other hand, several studies revealed a strong association between different variants within the *FTO* gene and BMI or diabetes supporting that the impact of *FTO* on obesity or diabetes is population-dependent [29–32]. A recent study conducted by Wang and colleagues [33] showed that *FTO* protein expression in T2DM patients was higher than in healthy controls which was positively correlated with T2DM severity, BMI and waist circumference.

Our study revealed that, among all study subjects, the TT carriers had lower BMI compared to AT and AA carriers (unadjusted *p* = 0.03) but when the mean BMI was stratified by glycemic status across *FTO* genotypes, the association with BMI was lost in the control group (*p* = 0.7). In diabetic group, an additive trend of the allele A with increased BMI was observed but was not significant (*p* = 0.057). However, further studies with larger sample size and greater statistical power are needed to replicate these findings. In 2016, a study conducted on Egyptian children and adolescents didn’t show an association between the polymorphism rs9939609 and BMI. However, that study revealed a significant correlation between LDL and *FTO* rs9939609 supporting the idea that this variant can be a determinant of obesity due to its effect on the lipid profile [34].

The high prevalence of obesity among our diabetic and control group (64 and 44%, respectively) could be attributed to other variants within *FTO* and or other genes which can be modulated by environmental factors and lifestyle. Anyhow, as our diabetic cases were older and had higher BMI than controls, we adjusted for the possible confounding effect of age, sex, and BMI in all the logistic regression analyses while investigating T2DM risk across *FTO* genotypes. Recently, a study conducted by Celis-Morales et al. [35] reported that physical activity attenuates the effect of *FTO* on BMI. Another study conducted on Emirati people showed that the AA carriers who were physically active had a lower mean BMI than those who were physically inactive, while other studies conducted on African Americans and Europeans showed no such interaction [36, 37]. Furthermore, a recent cross sectional study in a multiethnic population suggested that high dietary protein intake may protect against the effects of risk variants in the *FTO* gene on BMI and waist circumference [28]. In this study and due to the lack of data regarding physical activity or diet intake, we were unable to examine the influence of physical activity /diet on the impact of *FTO* variant on BMI. Although *FTO* -T2DM association was found, the lack of association between *FTO* rs9939609 and obesity is most probably due to the small sample size -and thereby decreased statistical power- which was the most important limitation in this study and thus larger sample size is required to verify these results. However, weight, skinfold thicknesses, body fat percentage and waist circumference are reported to be more reliable markers of obesity than BMI [38]. We believe that obesity-related genetic variants also modulate glucose–insulin secretion. Therefore, leaner cases should be recruited while investigating gene–T2DM association among Palestinians.

On the other hand, we did not find any association of *FTO* rs9939609 with the T2DM complications and the prevalence of CVD among the studied population. This is consistent with recent findings showing no association of *FTO* rs9939609 variant with diabetic retinopathy and nephropathy [39]. However, a meta-analysis study reported significant association of the *FTO* rs9939609 variant with CVD risk, which was independent of BMI and other conventional CVD risk factors [40].

## Conclusion

The *FTO* rs9939609 variant was significantly associated with T2DM in Palestine. However, further analysis with larger sample size and data on physical activity and diet intake is required to elucidate the role of this variant and other variants of *FTO* gene on the predisposition to increased BMI in Palestinians.

## Abbreviations

BMI: Body max index
CVD: Cardiovascular disease
*FTO*: Fat-mass and obesity-associated gene
GWAS: Genome-wide association
OR: Odd ratio
PCR: Polymerase chain reaction
RFLP: Restriction fragment length polymorphism
T2DM: Type 2 diabetes mellitus
*TCF7L2*: Transcription factor 7 like 2
WHO: World Health Organization
